# How Institutions Can Protect the Mental Health and Psychosocial Well-Being of Their Healthcare Workers in the Current COVID-19 Pandemic

**DOI:** 10.6061/clinics/2020/e1963

**Published:** 2020-05-25

**Authors:** Pedro Fukuti, Caroline Louise Mesquita Uchôa, Marina Flaborea Mazzoco, Felipe Corchs, Carla Satie Kamitsuji, Luciane De Rossi, Izabel Cristina Rios, Selma Lancman, Eloisa Bonfa, Tarcisio Eloy Pessoa de Barros-Filho, Eurípedes Constantino Miguel

**Affiliations:** I Faculdade de Medicina FMUSP, Universidade de Sao Paulo, Sao Paulo, SP, BR.; II Hospital das Clinicas HCFMUSP, Faculdade de Medicina, Universidade de Sao Paulo, Sao Paulo, SP, BR.

When the World Health Organisation (WHO) declared the spread of the severe acute respiratory syndrome-coronavirus 2 (SARS-CoV-2) a global pandemic on March 11, 2020, there were approximately 147,000 confirmed cases worldwide. Just one month later, the COVID-19 disease had spread dramatically; the number of cases had increased ten-fold (1), with 15% of infected patients requiring hospitalization and 5% in intensive care units (3). This meteoric rise in cases resulted in an overloaded demand for medical resources, often exceeding the available resources of healthcare systems around the world.

Although we often conceive of healthcare systems in terms of the physical hospital beds and medical equipment, the system’s most fundamental, valuable, and vulnerable assets are, undoubtedly, its manpower resources. Medical doctors, nurses, physiotherapists, technicians, and countless other professionals must all come together for the system to work effectively. Indeed, in order to buffer the effect of the pandemic on our healthcare systems and society as a whole, we rely heavily on the extent to which these individuals can function in a cohesive, effective manner (5).

Healthcare workers are being impacted by the current pandemic on two fronts. Like all of us, they are navigating social distancing, school and day care closures, the economic crisis, concerns about the health of their loved ones, and general uncertainty about the future. Also, because of their profession, they are likely to be exposed to overloaded working hours and a higher risk of infection amidst potential shortages of adequate personal protection equipment (PPE) and other supplies. Further stressful factors include feeling a lack of control or sense of helplessness, daily contact with suffering and death, as well as the need to communicate bad news and establish new communication strategies with family members who cannot visit hospitalized patients. In addition, concerns about infecting their families has resulted in many professionals leaving their homes and sheltering elsewhere, which may further worsen their psychological well-being. Finally, they are worried about whether they will be prioritized in care if they become ill and whether they will face ethical dilemmas such as those reported in other countries where the health system has collapsed (7).

Clearly, this pandemic is exerting great stress on the personnel working on the front line of efforts to control the virus in the healthcare system (5). Studies have already shown that most health care providers are exhibiting stress-related symptoms such as anxiety, depression, sleep disturbances, and emotional distress, and around 50% of them will fulfil criteria for a mental disorder (6). It is still not completely clear who among us is at a higher risk. As an example, early data suggest that females nurses may be particularly vulnerable (6,8), especially those working in direct contact with infected patients for longer hours. Also, anyone with a pre-existing chronic disease or mental health condition is at greater risk (5,6).

These data demonstrate that we are facing a situation in which the backbone of our health system, our personnel, is highly exposed, both physically and psychologically. The relevance of that unfolds in the consequences: if care providers are hampered by mental health and psychosocial issues, infection rates will increase (due to lower compliance with safe practices), which, in turn, would reduce staff numbers and amplify emotional distress in a vicious cycle. Indeed, this pandemic has already resulted in soaring rates of absenteeism, medical leaves, and even resignations.

For these reasons, any strategy to combat the COVID-19 crisis must take the mental health and psychosocial aspects of its healthcare workers into consideration. The strategy should be established at several levels: governmental, institutional, and individual. In fact, recent studies show that governmental and institutional attitudes toward the pandemic can directly increase motivation and performance levels of healthcare personnel, thereby protecting against negative mental health effects (8). Simply put, our healthcare professionals must feel that the government and medical institutions understand how stressful the current situation is and that they are taking actions to take care of them. This includes clear communication with the staff, provision of protective measures and PPEs, and sensitive administration of work shifts. Governments should also establish public policies that allow institutions to guarantee health care assistance, social (e.g. childcare needs, paid time off) and financial support to the health care professionals and their families. In addition, open access to mental and psychosocial support and treatment is critical and must be taken into account.

To address the aforementioned issues, the University of São Paulo School of Medicine and its Health Complex, Hospital das Clínicas, developed the program “COMVC19: The Mental Health and Psychosocial Well-Being Personal Protective Equipment to the Health Professionals involved in the Combat against the COVID-19 Pandemic.” This program is designed to offer mental health and psychosocial support and psychological/psychiatric treatment to approximately 20,000 hospital employees. It has been officially broadcasted to members of the whole complex through electronic means (a link to the webpage) and new information is continuously updated. Based on what we have learned from our interactions with our professionals, teams, and their leadership, the program comprises three branches: mental health and psychosocial support, education, and research.

The composite term ‘mental health and psychosocial support’ (MHPSS) is used in the Inter-Agency Standing Committee (IASC) Guidelines for MHPSS in Emergency Settings to *describe “any type of local or outside support that aims to protect or promote psychosocial well-being and/or prevent or treat mental health condition”*. The global humanitarian system uses the term MHPSS to unite a broad range of actors responding to emergencies such as the COVID-19 pandemic, including those working with biological approaches and sociocultural approaches in health, social, education, and community settings, as well as to “*underscore the need for diverse, complementary approaches in providing appropriate support”* (4). The IASC Guidelines for MHPSS in Emergency Settings recommends that multiple levels of interventions be integrated within outbreak response activities. These levels align with a broad spectrum of mental health and psychosocial needs and are represented in a pyramid of interventions (Figure 1) ranging from embedding social and cultural considerations in basic services to providing specialized services for individuals with more severe conditions. Core principles include: do no harm, promote human rights and equality, use participatory approaches, build on existing resources and capacities, adopt multi-layered interventions, and work with integrated support systems (4). The MHPSS branch of the COMVC19 program (Figure 1) consists of coordinated measures that range from preventive actions and therapeutic interventions to rehabilitation, if required. Secondary prevention encompasses the training of members of our medical units following the “Psychological First Aid” principles (2), as well as support groups for professionals working on the frontline - hence, those who are more likely to develop mental disorders (6). This means that individuals who require mental health support will have access to psychological groups acting locally within their wards or to a hotline held by supervised residents of psychiatry on call, 24/7. In both cases, mental health professionals will provide empathic listening in the short term. This support stage will allow us to identify individuals who require referral to psychiatric and/or psychological (brief psychotherapy) treatments, or a psychiatric ER if the situation constitutes an emergency. Finally, specific occupational therapy will be provided for those in quarantine or on medical leave.

The educational branch of the initiative focuses on the training of residents in all these actions and will include six hours of short video-classes prepared by our group of experienced assistants. Critically, these videos will target both health professionals and the general public, thereby scaling up the impact of the program. They are freely available online (https://sites.google.com/hc.fm.usp.br/comvc-19/comvc-19),

Finally, a research branch was developed to monitor our employees' mental health regularly through an online platform. Our aim is to investigate and identify risk and devise protective interventions for specific groups. Further, using standard instruments, each intervention will be evaluated in real time. Hereafter, the analyses, interpretation, and publication of these experiences will allow us to improve the whole program, as well as share our failures and successes with the scientific community.

Specialists estimate that the world will endure a long battle against the COVID-19 pandemic and its consequences. To ensure success, it is essential to keep our healthcare workers active, motivated, and healthy. Thus, we hereby recommend that all health institutions pay special attention to the mental health and psychosocial well-being of their workers. If our frontline health soldiers should suffer due to mental health and psychosocial burdens, all our other weaponry will be compromised and the war will be lost. These actions will mitigate a second wave of high incidence of mental health and psychosocial problems as a sequelae of this pandemic that could otherwise be prevented by these interventions.

## AUTHOR CONTRIBUTIONS

Fukuti P, Uchôa CLM and Miguel EC were responsible for the manuscript original draft. Mazzoco MF, Corchs F, Kamitsuji CS, Rossi L, Rios IC, Lancman S, Bonfa E and Barros-Filho TEP were responsible for the manuscript writing, editing and review.

## Figures and Tables

**Figure 1:**
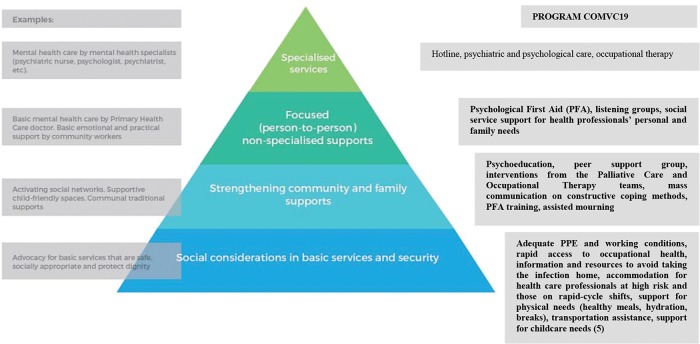
Intervention pyramid for mental health and psychosocial support.
